# On the HKUST-1/GO and HKUST-1/rGO Composites: The Impact of Synthesis Method on Physicochemical Properties

**DOI:** 10.3390/molecules27207082

**Published:** 2022-10-20

**Authors:** Paulina Jagódka, Krzysztof Matus, Agata Łamacz

**Affiliations:** 1 Department of Engineering and Technology of Chemical Processes, Wrocław University of Science and Technology, Gdańska 7/9, 50-344 Wrocław, Poland; 2 Materials Research Laboratory, Silesian University of Technology, 18A Konarskiego, 44-100 Gliwice, Poland

**Keywords:** HKUST-1, GO, Cu/rGO, composite morphology, textural properties, synthesis parameters

## Abstract

The chemical stability and adsorptive/catalytic properties of the most widely studied metal–organic framework (MOF), which is HKUST-1, can be improved by its combination with graphene oxide (GO) or reduced graphene oxide (rGO). The chemistry of GO or rGO surfaces has a significant impact on their interaction with MOFs. In this work, we demonstrate that GO and rGO interaction with HKUST-1 influences the morphology and textural properties but has no impact on the thermal stability of the final composites. We also show that synthesis environment, e.g., stirring, to some extent influences the formation of HKUST-1/GO and HKUST-1/rGO composites. Homogeneous samples of the sandwich-type composite can be obtained when using reduced graphene oxide decorated with copper (Cu/rGO), which, owing to the presence of Cu sites, allows the direct crystallisation of HKUST-1 and its further growth on the graphene surface. This work is the first part of our research on HKUST-1/GO and HKUST-1/rGO and deals with the influence of the type of graphene material and synthesis parameters on the composites’ physicochemical properties that were determined by using X-ray diffraction, scanning and transmission electron microscopy, Fourier transform infrared spectroscopy, X-ray photoelectron spectroscopy, and thermogravimetric analysis.

## 1. Introduction

A growing interest in metal–organic frameworks (MOFs) results from their unique physicochemical properties, such as the organic–inorganic nature, crystalline structure, outstanding specific surface area (SSA), stable porosity, and the infinite possibility of implementing chemical modifications [1,2,3,4]. MOFs find application in many areas, including adsorption and separation processes, drug delivery, detection of gaseous and liquid compounds, energy storage, water treatment, or catalysis. However, the practical application of some MOFs may be cut down owing to their reduced chemical or thermal stability [2,5,6]. Popular MOFs, such as HKUST-1 [7], MIL-101 [8], and UiO-66 [9], are reported to be thermally stable up to 240, 275, and 540 °C, respectively. However, there is often a discrepancy between different reports on the same MOF structures, which usually arises from different synthesis parameters. For example, the stability of the HKUST-1 framework to 250 °C was reported in [10] and to 310–330 °C in [11]. In addition, the prolonged exposition to increased temperature (but in the theoretical range of MOF stability) may result in the slow decomposition of the linker molecules, and finally, the collapse of the framework. Some MOFs that have the potential to be used as adsorbents or catalysts (thanks to their textural properties, porosity, and surface chemistry) are unstable in the conditions of the given process and decompose. For example, HKUST-1, which could be used in the adsorption of small particles, is unstable in the presence of ammonia [12]. This MOF is also known for its hydrophilicity. In the presence of moisture, the Cu^2+^ coordinatively unsaturated sites are easily occupied with water molecules, which decreases the adsorptive and catalytic properties of the material. The prolonged exposure to humidity can damage the crystalline structure of HKUST-1 because water that condensates inside the cavities can hydrolyse the Cu-O bond [13,14].

To overcome these shortcomings and to improve MOFs performance, they may be modified in the nodes or linkers, doped with the additional metal ions, grafted with the functional moieties, or combined with other materials to give composites. MOFs have already been merged with metals nanoparticles (NPs) [15], silica [16], organic polymers [17], or carbon [1,18,19]. The latter, i.e., MOF composites with activated carbons (ACs), carbon nanotubes (CNTs), graphene oxide (GO), graphite, or fullerenes (all occurring in the form of powder, fibres, monoliths, etc.), have been the most widely studied [19]. Carbon materials are characterised by a high Young’s modulus, excellent chemical and thermal resistance, and outstanding optical and electronic properties, thus improving stability, conductivity, and adsorptive/catalytic properties of MOFs [2]. 

HKUST-1, first described by Chui et al. [7] in 1999, has been the most studied MOF. In its framework, the Cu^2+^ ions are connected to oxygen atoms in 1,3,5-benzenetricarboxylic ions to form dimeric Cu paddle-wheel units with available coordination sites on each metal atom. Due to the presence of coordinatively unsaturated metal sites (CUMSs), HKUST-1 is widely used in adsorption or catalysis [20,21]. On the other hand, the interactions of CUMSs with H_2_O molecules make HKUST-1 unstable in the presence of moisture, which limits its applicability [22]. However, studies show that the unsaturated Cu-H_2_O interaction can be significantly attenuated by the introduction of graphene oxide (GO) [12,18]. GO is a two-dimensional nanomaterial of a honeycomb structure formed of carbon atoms. It contains oxygen-based functional groups, e.g., hydroxyl (-OH), carbonyl (C=O), alkoxy (C-O-C), and carboxyl (-COOH). Owing to the presence of functional groups, GO is prone to oxidation even at mild temperatures, unlike graphene, which is characterised by high thermal stability and mechanical strength. Oxygen moieties present on the graphene surface are the centres for nucleation and growth of metal nanoparticles (NPs) [23,24]. They can also react with metal ions in MOFs. The formation of MOF-GO hybrids occurs through coordination bonds between the metal in MOF and the oxygen in GO, which makes the hybrid stable. Combining HKUST-1 with GO may prevent decomposition of the former in water and improve its adsorptive, photochemical, and electrical properties [23]. Moreover, GO can significantly affect the textural properties of HKUST-1 as new pores may form between both components [1]. 

A pioneering study on HKUST-1/GO composites was published by Bandosz et al. [25]. It was proven that the arrangement of GO and HKUST-1 depends on the type of functional groups on the GO surface, involved in the formation of the composite, and the geometry of the metal nodes in HKUST-1 [1,19]. Moreover, the significant influence of GO concentration on the textural properties of HKUST-1/GO was observed. The composites obtained by Bandosz’s group were successfully used in the reactive sorption of small particles, i.e., NO_2_ [26], NH_3_ [12,25,27], H_2_S [27,28], as well as the physical adsorption of CO_2_ [29] under ambient conditions, proving the synergistic effect of the composite components resulting in increased adsorptive properties. A significant influence of the presence of functional moieties on the GO surface was also reported by Al-Naddaf et al. [30], who synthesised HKUST-1 composites with pristine, reduced, and COOH-functionalised GO via hydrothermal synthesis. The obtained composites demonstrated different physicochemical properties (also in terms of CH_4_ sorption capacity) probably due to the presence of new pores in HKUST-1/rGO, as well as the synergistic interaction of MOF and defective rGO sheets. Compared to HKUST-1 alone, the HKUST-1/GO composite (obtained via in situ solvo- or hydrothermal synthesis, with or without stirring) shows improved performance in gas separation [31], adsorption of molecules from aqueous solutions [32,33], energy storage [34], and catalysis [35]. Recently, attempts have been made to modify the synthesis of MOF-GO or MOF-graphite oxide composites. For example, HKUST-1/GO was synthesised via a Pickering emulsion system, in which the MOF is produced at the interface of GO Pickering emulsion droplets at 60 °C for 60 min [36]. The columnar rod-shaped composites obtained in this way have a microporous structure and a high sorption capacity towards CO_2_. The HKUST-1/GO aerogels formed in supercritical CO_2_ synthesis also reveal excellent CO_2_ and CH_4_ adsorption and separation abilities [37]. The composites can be obtained in situ, i.e., from HKUST-1 precursors and GO solution, as well as ex situ, from previously prepared HKUST-1 and GO suspensions. The synthesised aerogels show a hierarchical structure with macro-, meso-, and micropores. Another procedure of HKUST-1/GO synthesis is a fast method based on anion exchange [38]. In this method, ZnO reacts with Cu(NO_3_)_2_ to form hydroxy double salt (Zn,Cu)(OH)NO_3_, which is converted to HKUST-1 [39]. Although the synthesis of the composite was successful, its textural properties were slightly worse than those of HKUST-1/GO obtained via the hydrothermal method. In addition, no improvement in sorption properties towards SO_2_ was noticed for this material [38].

In this work, the HKUST-1/GO composites were obtained in the solvothermal synthesis using pristine GO or GO pre-decorated with Cu nanoparticles (Cu/rGO). The physicochemical features of the obtained composites, such as crystallographic and textural properties, morphology, chemical composition, and thermal stability, were studied in terms of the synthesis parameters and the type of used graphene material. The formation of HKUST-1/GO composites typically involves the reaction between Cu ions in HKUST-1 and the functional groups (e.g., carboxyl, epoxy, or hydroxide) that are present on the surface and edges of GO [1,19]. The enhancement of the formation of homogeneous composite may be achieved by using the Cu-pre-decorated graphene oxide because anchoring the CuNPs on the surface of GO allows the creation of well-dispersed sites for HKUST-1 crystallisation. In this approach, GO is reduced to rGO—first, because positively charged Cu^2+^ ions adsorb on the negatively charged surface of graphene oxide and, thus, “consume” the functional groups; secondly, the process of CuNPs formation is conducted in the presence of hydrazine, which, except from reducing Cu species to Cu^0^, may also reduce the unoccupied functional groups on GO [39]. 

## 2. Results and Discussion

### 2.1. Crystallographic Properties of HKUST-1/GO and HKUST-1/rGO Composites

**Figure 1:**
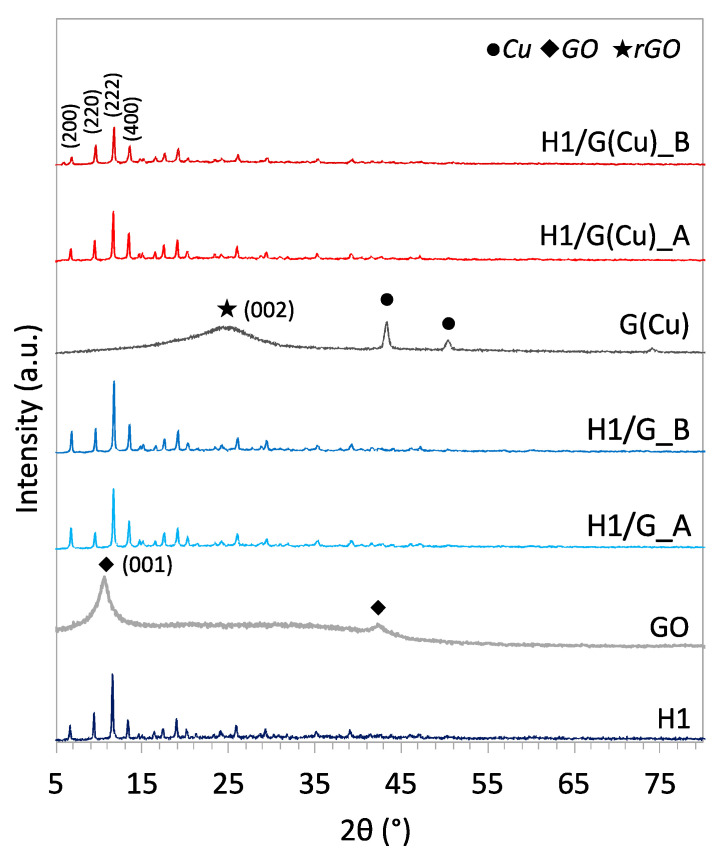
XRD patterns of the HKUST-1/GO composites (H1/G_A, H1/G_B, H1/G(Cu)_A, and H1/G(Cu)_B) and their components alone: HKUST-1 (H1), GO, and Cu/rGO.

The XRD patterns of pristine HKUST-1 (denoted H1), graphene oxide (GO), and GO pre-decorated with copper nanoparticles (denoted Cu/rGO) are displayed in Figure 1 together with their composites obtained via conventional solvothermal synthesis (assigned A) and the one assisted with stirring (ascribed B). On the diffractogram for H1, one can observe the diffraction peaks at 2θ = 6.6, 9.4, 11.5, and 13.4° corresponding to the (200), (220), (222), and (400) crystal planes [40]. The XRD plot for pristine GO shows a single broad peak at 10.74°, which is assigned to an (001) inter-planar spacing of 0.823 nm. On the diffractograms of H1/G_A and H1/G_B, one can observe the reflexes coming from the HKUST-1 phase only, while the peaks of the graphene material (GO or rGO) are not visible (likewise reported in [1]). This can be explained by a very good dispersion of the graphene phase in the composites. It must be noted that after the synthesis, the free oxygen groups are removed from GO; hence, in the composite, it is occurring in its reduced state.

Pre-decoration of GO with CuNPs aimed at providing the adsorption sites for BTC^3−^ and initialisation of the crystallisation of HKUST-1 directly on the carbon support. As the Cu deposition on GO was conducted in the presence of hydrazine, the oxygen-containing groups on the surface of GO were reduced. This is proven by the presence of the diffraction peak at 2θ = 24.4° that corresponds to the (002) plane in the reduced graphene oxide (rGO) and the inter-planar spacing of 0.366 nm [41]. In addition, the diffractogram of Cu/rGO exhibits reflexes at 2θ = 43.4, 50.5, and 74.1° that are assigned, respectively, to (111), (200), and (220) crystalline planes in the zero-valent copper [42]. These reflexes are not observed on the XRD plot for H1/G(Cu) samples, indicating that Cu was engaged in the formation of the connection between HKUST-1 and rGO. 

Table 1 shows the average sizes of HKUST-1 and Cu crystallites (D) calculated from the Scherrer equation (one must remember that it allows only a rough estimation of the crystal size). Based on obtained results, it is noticed that in the H1 sample, the mean sizes of HKUST-1 crystallites in directions perpendicular to the (200), (220), and (222) planes are 48, 56, and 52.9 nm, respectively. These values are only slightly lower for H1/G and H1/G(Cu) composites. Hence, combining HKUST-1 with the graphene component (GO or Cu/rGO) via solvothermal synthesis insignificantly decreases the size of MOF crystallites. Comparing the sizes of HKUST-1 crystallites in composites obtained in static solvothermal synthesis (method A), one observes that they are slightly bigger when this MOF is composed with Cu/rGO than with GO. The opposite is observed when stirring is applied (method B). Stirring somewhat increases the size of HKUST-1 crystallites in the composite with pristine GO and decreases it in the composite with Cu/rGO.

### 2.2. Morphology of HKUST-1/GO and HKUST-1/rGO Composites

**Figure 2:**
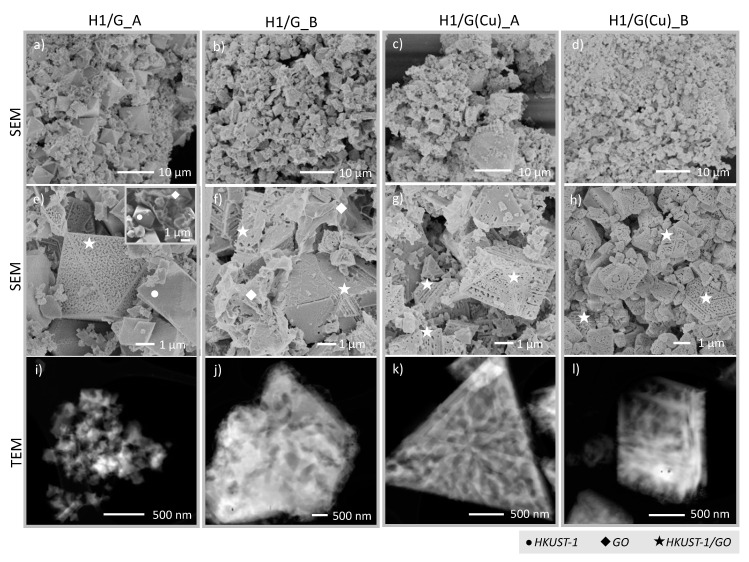
SEM (**a**–**h**) and TEM images (**i**–**l**) of HKUST-1/GO composites: H1/G_A (**a**,**e**,**i**), H1/G_B (**b**,**f**,**j**), H1/G(Cu)_A (**c**,**g**,**k**), and H1/G(Cu)_B (**d**,**h**,**l**).

The morphology of the obtained composites and their components alone was studied by scanning electron microscopy (SEM) and transmission electron microscopy (TEM). From SEM (Figure 2a–h), it is observed that regardless of the synthesis method, the particles of all composites are octahedral in their shape, which is typical for HKUST-1 [43]. The particles are porous and their sizes range from 0.2 to 10 µm. The homogeneity of the obtained materials is affected both by the synthesis method applied and the type of graphene component used (GO or Cu/rGO). Concerning the particle size, the samples obtained under continuous stirring (H1/G_B and H1/G(Cu)_B) are more uniform than those obtained in conventional, static experiments (Figure 2a–d). By providing even heat distribution and mass transfer, stirring affects the rates of nucleation and crystal growth of HKUST-1. Moreover, stirring provides an even distribution of the graphene component in the reaction mixture for HKUST-1 crystallisation; thus, the obtained composites (H1/G_B and H1/G(Cu)_B) are more homogeneous in their morphology than those synthesised in static conditions. Figure 2a,e prove the existence of three phases in the H1/G_A sample: HKUST-1, GO, and the HKUST-1/GO composite. Application of stirring (sample H1/G_B, Figure 2b,f) helps to form composite particles, but still some loose GO plates are observed between them. Using the pre-decorated graphene component (Cu/rGO) facilitates the formation of the composite because HKUST-1 crystallisation starts directly on the Cu sites that are very well dispersed on the graphene surface.

**Figure 3:**
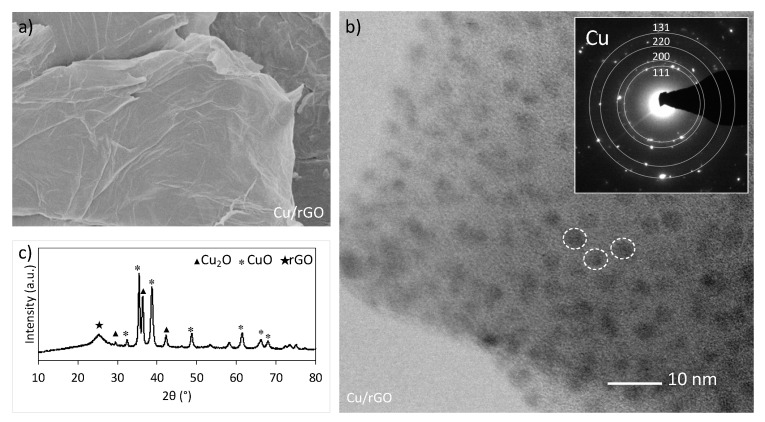
SEM (**a**) and TEM with SAED of CuNPs (**b**) of Cu/rGO used for synthesis of H1/G(Cu) composites. The XRD pattern of Cu/rGO after heating at 120 °C/2 h in DMF and air (**c**).

**Figure 4:**
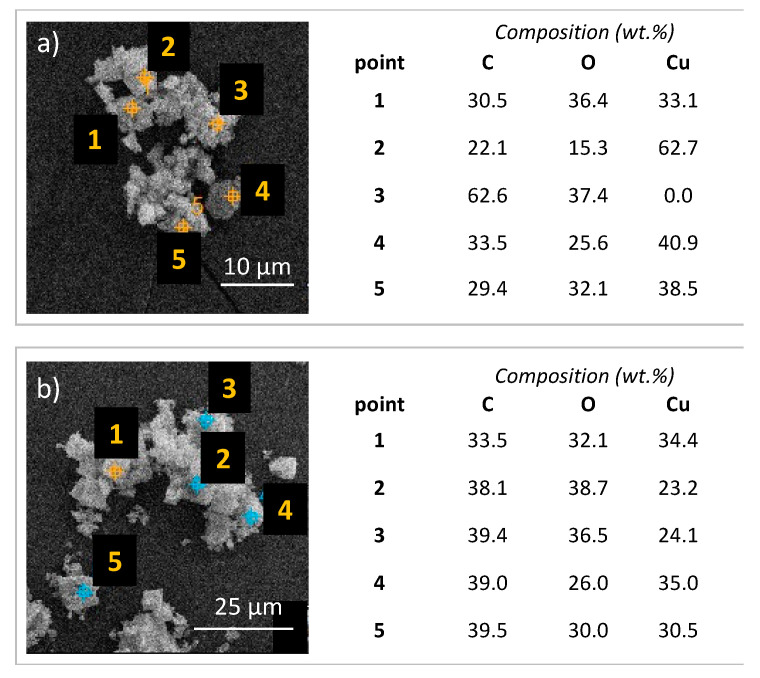
Composition of the H1/G_B (**a**) and H1/G(Cu)_B (**b**) composites determined by EDS in selected points of the samples.

Figure 3b shows highly dispersed CuNPs of 2–4 nm in diameter but the SEM images—not shown—indicate that Cu exists also in the form of bigger agglomerates, thus explaining why the mean size of Cu crystallites calculated from XRD (Table 1) shows the higher value (17 nm). In the Cu/rGO, copper is in its reduced state as was previously demonstrated by the XRD (Figure 1) and SAED (Figure 3b). However, knowing that CuNPs show instability in the oxidative atmosphere and under increased temperature (HKUST-1/rGO was synthesised at 120 °C in DMF and in the presence of air), it was assumed that prior to HKUST-1 crystallisation, the oxidation of Cu^0^ to Cu^+^ or Cu^2+^ took place. The blank experiment, in which Cu/rGO was subjected to heating at 120 °C for 2 h in DMF and in the presence of air, confirmed that assumption. The XRD analysis of Cu/rGO after a blank test (Figure 3c) revealed CuNPs oxidation to Cu_2_O and CuO. Hence, in the real synthesis, copper oxide reacts with H_3_BTC to form copper carboxylate of the dimeric Cu^II^ acetate-like structure [44]. HKUST-1 then grows on the graphene surface by repeated addition of the copper acetate followed by trimesate ions [45]. Using Cu/rGO in the syntheses of composites provides total consumption of the graphene component. The SEM images of H1/G(Cu)_A and H1/G(Cu)_B show that the obtained samples are homogenous. The EDS analyses performed for H1/G_B and H1/G(Cu)_B (Figure 4) proved that the distribution of C, O, and Cu was better in the latter. From SEM, both samples show good homogeneity owing to the application of Cu/rGO. However, the EDS results prove our hypothesis that stirring enhances the formation of the connection between HKUST-1 and graphene material.

### 2.3. Surface Chemistry of HKUST-1/GO and HKUST-1/rGO Composites

**Figure 5:**
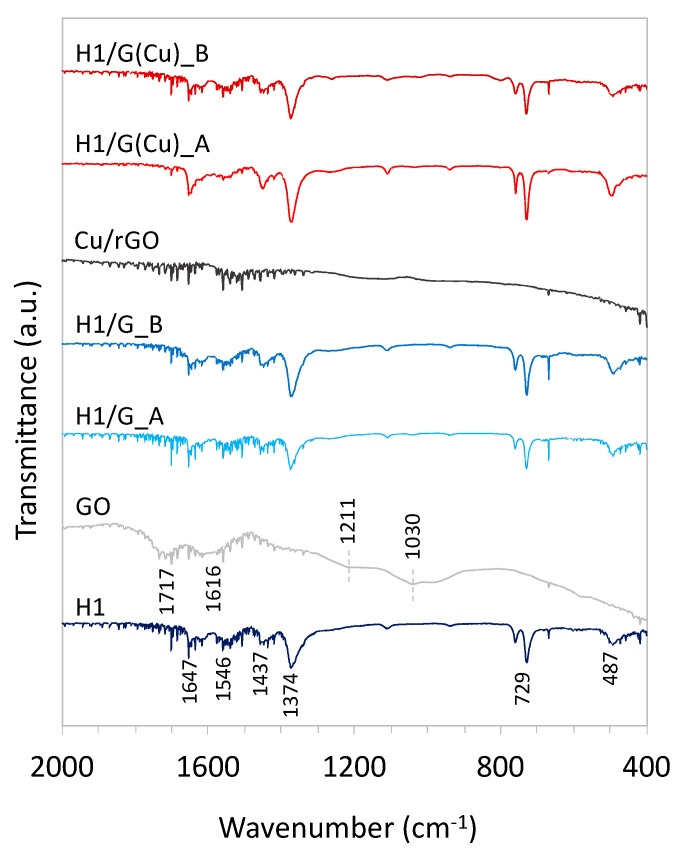
FTIR spectra of HKUST-1/GO composites and their components collected in 2000–400 cm^−1^ range.

The composites and their components alone were studied by FTIR spectroscopy in the 4000–400 cm^−1^ range. Obtained spectra (reduced to 2000–400 cm^−1^ region) are shown in Figure 5. The spectrum of the H1 sample shows typical vibration frequencies for chemical bonds in HKUST-1 [1,46]. The bands at 1647 and 1437 cm^−1^ are assigned to the asymmetric (*ν*_as_) and symmetric (*ν*_sym_) vibrations of the carboxylate groups, respectively. Bands at 1546 and 1374 cm^−1^ are assigned to stretching vibrations of C=C, confirming the presence of BTC^3−^ linkers [46]. Bands at 487 and 729 cm^−1^ correspond to Cu-O bonds through which Cu^2+^ ions are coordinated with the carboxylate groups of the ligands [47,48]. The FTIR spectra of HKUST-1/GO composites show the bands at the same frequencies as observed for H1. The absence of bands at 1193, 1232, and 1710 cm^−1^ coming from C-O stretching, OH bending, and C=O stretching speaks for the absence of free carboxylic groups in the samples. In the case of composites, such a situation is expected because HKUST-1 and graphene material form sandwich-type structures; hence, the terminal carboxylic groups in MOF are occupied by the graphene component.

FTIR is not the best technique for characterisation of carbon materials, but it often allows the surface functional groups in those materials to be determined. On the spectra of the GO, one can observe broad bands at 1030, 1211, 1616, and 1717 cm^−1^ that can be assigned to the C-O, -OH, and -COOH species [48]. The spectrum of Cu/rGO does not display intensive bands.

**Figure 6:**
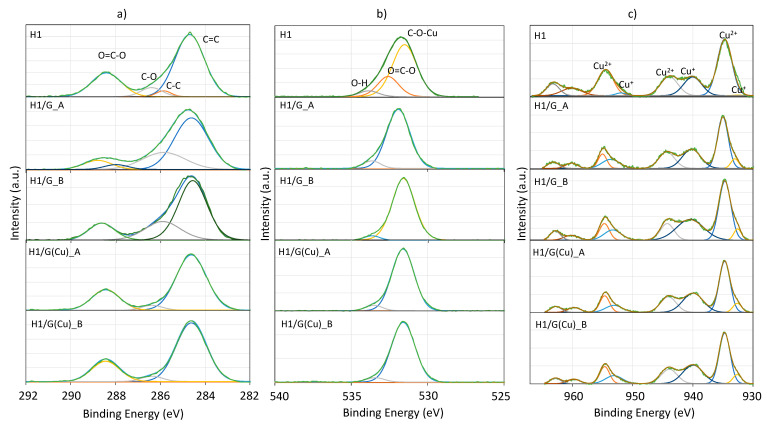
The C 1s (**a**), O 1s (**b**), and Cu 2p (**c**) XPS spectra for H1, H1/G, and H1/G(Cu) composites.

Determination of the electronic properties of HKUST-1 and obtained composites was performed with the XPS (Figure 6). The deconvolution of high-resolution C 1s, O 1s, and Cu 2p spectra was performed to compare the chemical states of C, O, and Cu in the pristine HKUST-1 and its composites with GO and Cu/rGO. The C 1s spectra show peaks at binding energies (B.E.) of ca. 284.6, 285.4, 286.6, and 288.8 eV, corresponding to C=C, C-C, C-O, and O=C-O bonds, respectively. These peaks are characteristic for the trimesic acid in HKUST-1 [49], as well as for GO and rGO [50] (not shown in this work). The peak at ca. 285.4 eV can also be ascribed to C-N whose presence in the samples may arise either from the reduction of GO with hydrazine (N_2_H_4_) or DMF, which can still be present in the pores of HKUST-1 [51]. From Figure 6 and Table 2, it is noticed that the concentration of oxygen-containing groups is lower for the composites obtained with Cu/rGO. The C 1s spectra of H1/G(Cu)_A and H1/G(Cu)_B show a lower contribution of C-O bonds (peak at B.E. ~ 286.4 eV). Moreover, the absence of a peak at ca. 289.4 eV related to O=C-OH proves that in studied materials, there are no free carboxylic groups coming either from BTC^3−^ in HKUST-1 or from the surfaces of GO or Cu/rGO (hydrazine does not reduce carboxylic groups on GO [52]), because these groups were employed in the formation of the HKUST-1/graphene composite.

The deconvoluted O 1s spectra show three oxygen species: the C−O−Cu in the paddle-wheel SBU in HKUST-1 (B.E. ~ 531.5 eV), the O-C=O of the trimesic acid (B.E. ~ 532.6 eV), and the C-O-H (~533.8 eV) [53,54]. The significant contribution of C-O-Cu in the H1/G and H1/G(Cu) samples proves the creation of the composite.

The Cu 2p XPS spectra consist of a few peaks indicating the co-existence of Cu^2+^ and Cu^+^. The Cu 2p_3/2_ and Cu 2p_1/2_ peaks of Cu^2+^ are situated at ca. 934 and 954 eV, respectively, with a shake-up satellite at ca. 944 eV, while the peaks of Cu^+^ are observed at ca. 932 and 952 eV with a shake-up satellite at ca. 940 eV [55]. From all spectra, the domination of the divalent Cu is observed. The presence of Cu^+^ ions can be attributed to oxide impurities or —as has been reported by Szanyi et al. [56]—can arise from the redox treatments, using vacuum and/or reducing gases. The Cu^2+^/Cu^+^ ratio presented in this work HKUST-1 is 4.1, while for the composites, it ranges from 1.3 to 1.7; hence, the contribution of reduced copper species increases, which can be owed to the opening of the paddle-wheel Cu_2_(CO_2_)_4_ units being in contact with the graphene component.

### 2.4. Thermal Stability and Composition of Composites

**Figure 7:**
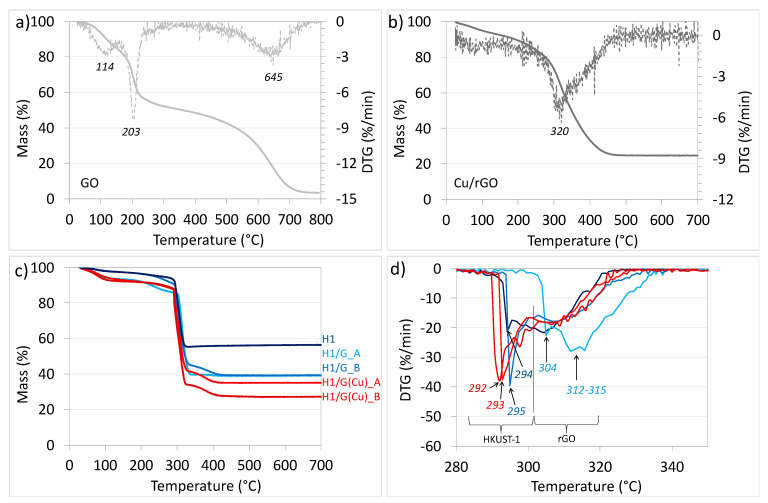
TG (solid line) and DTG (dotted line) for GO (**a**) and Cu/rGO (**b**). The collective TG (**c**) and DTG (**d**) for H1, H1/G_A, H1/G_B, H1/G(Cu)_A, and H1/G(Cu)_B.

The composites and their components alone were subjected to thermogravimetric analysis (TGA) to determine the quantitative contribution of each phase and the thermal stability of the materials. Figure 7a shows the TGA results that allow qualitative and quantitative determination of the GO composition. The DTG plots generated from TG curves reveal three main DTG peaks related to three mass loss occurrences: the first mass decrease of 14.6% is observed from RT to 150 °C and is assigned to evaporation of moisture; the second, rapid mass loss of 28.9% (from 150 to 237 °C) with a minor mass loss of 4.6% up to 307 °C is assigned to the removal of oxygen functional groups; while the third mass decrease (48.68%) with a maximal rate at 645 °C arises from oxidation of the carbon framework. The DTG plot obtained from the TG profile of Cu/rGO (Figure 7b) displays only one peak with a maximum at 320 °C, which is ascribed to the oxidation of carbon. The combustion process is finished at 480 °C. The residue (24.8%) is CuO. Comparing the TG/DTG plots for GO and Cu/rGO, one can conclude that the presence of Cu shifts the maximal rate of rGO mass loss to lower temperatures. CuNPs are known for a high surface activity that makes them susceptible to oxidation in air. The activation energy of bulk Cu oxidation is somewhat higher than for CuNPs [57,58]; nevertheless, both types of Cu particles can be oxidised at relatively low temperatures. It was reported by Yabuki and Tanaka [59] that rapid oxidation of CuNPs to Cu_2_O and CuO occurred from above 200 °C, while Nascimiento et al. [60] observed that CuO_x_ deposited on rGO decreased the temperature of rGO oxidation.

The combined TG and DTG plots for H1 and its composites with graphene materials are presented in Figure 7c,d. It can be observed that all materials reveal two stages of mass loss. The slow one observed up to ca. 290 °C arises from moisture removal, while the rapid one that finishes between 300 and 400 °C is related to (i) the destruction of the HKUST-1 framework due to combustion of organic linkers and (ii) oxidation of the graphene component. The DTG plots presented in Figure 7d indicate that HKUST-1 alone is stable up to ca. 292 °C and its stability is slightly increased to ca. 294 °C when it is combined with GO via solvothermal synthesis under continuous stirring (H1/G_B). A more important increase in thermal stability of HKUST-1 (up to ca. 302 °C) is observed in H1/G_A—the sample that, according to SEM, shows the less homogeneous morphology amongst all obtained composites. The composites obtained with Cu/rGO reveal the lowest thermal stability. Likewise, in the case of H1/G composites, application of stirring during synthesis results in less thermally stable material—the degradation of HKUST-1 in H1/G(Cu)_B starts below 290 °C. A higher thermal stability of composites obtained with GO compared to those obtained with Cu/rGO can arise from the bigger particle size in the former. As was evidenced by SEM (Figure 2), H1/G(Cu)_A and H1/G(Cu)_B are characterised with smaller particles, hence possessing more edge sites per mass unit and being more reactive in oxidative atmosphere. For combusting bigger particles, more energy is required.

**Figure 8:**
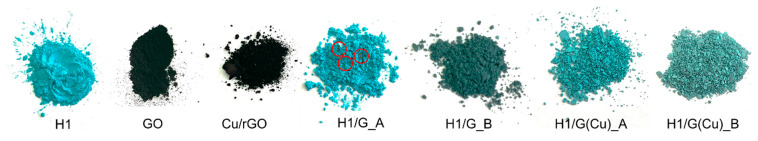
Pictures of HKUST-1/GO composites and their compounds alone. Red circles on the picture of H1/G_A indicate the black GO particles.

Table 3 shows a summary of the qualitative and quantitative composition of the obtained materials. As was indicated in Figure 7d, the mass decrease up to ca. 300 °C (first DTG peak) is linked with BTC^3−^ oxidation, while above this temperature, with the combustion of graphene (second, less intensive and broad DTG peak). The content of Cu in the samples was calculated from the residue CuO. It was calculated from the TG and DTG plots that the concentration of the graphene component in composites varies from 2 to 8.1 wt.%. The lowest amount of graphene is noticed for H1/G_A that, according to microscopic observations, shows the lowest homogeneity in terms of morphology—in this sample, part of HKUST-1 occurs as a free phase, unbonded with GO. Figure 8 shows the pictures of obtained samples. It is observed that the appearance (colour) of the H1/G_A sample is similar to pure HKUST-1, with visible black areas of graphene oxide (marked with red circles) proving poor integration of both components. It is expected that in this sample, the contribution of the composite phase is lowest among all prepared materials. Stirring improves the crystallisation of HKUST-1 directly on the surface of GO, and this increases the contribution of the GO phase in the composite (as was detected by TGA of H1/G_B). The concentration of the graphene component in the two composites obtained with Cu/rGO is similar. Hence, the application of stirring during synthesis does not influence the graphene contribution in those composites. Comparing the content of CuO (it is a residue after TGA) in materials obtained with GO and with Cu/rGO, one may notice that it is lower in the H1/G(Cu) samples.

### 2.5. Textural Properties of HKUST-1/GO and HKUST-1/rGO

**Figure 9:**
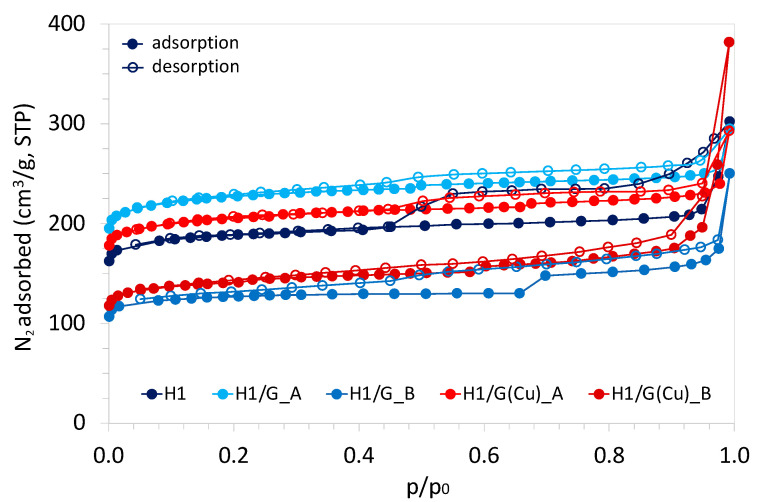
N_2_ adsorption–desorption isotherms at standard temperature and pressure (STP) for HKUST-1 and HKUST-1/GO composites; filled symbols stand for the adsorption; open symbols stand for the desorption.

The type I adsorption–desorption isotherms for H1, H1/G, and H1/G(Cu) composites that are presented in Figure 9 indicate that the obtained materials are microporous. However, the hysteresis loops observed at p/p0 > 0.4 suggest the presence of mesopores, which can be explained by the presence of empty spaces between particles. The BET surface area (S_BET_) of HKUST-1 obtained in this work is 666 m^2^/g (Table 4) (it usually ranges from 660 to 1700 m^2^/g [20,61]), while the S_BET_ of GO and Cu/rGO is 181 and 19 m^2^/g, respectively. Such a drastic drop in the surface area after GO decoration with Cu may be caused by the stacking of graphene sheets because of hydrazine treatment. The agglomeration of graphene sheets during GO reduction remains a major problem and has been reported, e.g., in [39,62]. On the contrary, the increase in BET surface area has been achieved when the GO reduction was conducted in the presence of surfactants [63], other reductants (such as thiourea dioxide [64]), or nitrogen [65]. Mohan et al. [66] studied the GO reduction with acids (HI, HBr, HCl, H_2_SO_4_, and C_6_H_8_O_6_), hydrazine, sodium borohydride, and dextrose, and, in all cases, observed the increase in S_BET_. Upon reduction, the functional groups in GO are removed, which increases the porosity of the resulting rGO. A similar situation is observed after deposition of NPs on GO [67].

The textural properties of composites obtained in this work depend on the method of their synthesis. The S_BET_ of the H1/G_A is 804 m^2^/g, while that of H1/G_B is only 450 m^2^/g. According to the results of TGA (Table 3), the H1/G_A sample contains the highest concentration of HKUST-1—being a high-surface-area compound—and the lowest concentration of GO, which is characterised by low S_BET_. Hence, a high contribution of free HKUST-1 in the H1/G_A (as was proven by SEM in Figure 2a,e) explains the highest S_BET_ of this sample. A significantly lower S_BET_ of H1/G_B can be due to the presence of a loose graphene phase occurring together with the composite phase (Figure 2b,f). From TGA, it is observed that the contribution of GO in this sample is higher than for H1/G_A. Both H1/G_A and H1/G_B differ in terms of the mean pore size. The H1/G_A has narrow pores of 2.3 nm such as the pore size in HKUST-1, while H1/G_B has pores of 3.4 nm such as in GO. In addition, the volume of micropores (V_micro_, Table 4) in H1/G_A is two times higher than in H1/G_B. It can be assumed that under continuous stirring, the GO flakes underwent agglomeration to some extent, which resulted in the decrease in surface area accessible for HKUST-1 crystallisation. The specific surface areas of composites obtained using Cu/rGO are lower than for the above-discussed H1/G materials. The H1/G(Cu)_A and H1/G(Cu)_B have S_BET_ values of 738 and 520 m^2^/g, respectively. Likewise, in the case of H1/G composites, the material obtained under continuous stirring (H1/G(Cu)_B) is characterised with a lower S_BET_ and lower volume of micropores than the composite synthesised in a static synthesis (H1/G(Cu)_A). This can be caused by the minor contribution of HKUST-1 in the latter (also proved by TGA, Table 3) that provides the microporosity and developed surface area to the composite.

## 3. Materials and Methods

### 3.1. Materials

The following reagents were used during the research: copper (II) nitrate trihydrate (Cu(NO_3_)_2_·3H_2_O, ≥99.0%, Sigma Aldrich, Poznań, Poland), copper acetate monohydrate (Cu(Ac)_2_·H_2_O, ≥99.0%, Merck, Poznań, Poland), 1,3,5-benzenetricarboxylic acid (H_3_BTC, 95%, Sigma Aldrich), hydrazine (NH_2_NH_2_·H_2_O, 64–65%, Sigma Aldrich, Poznań, Poland), dimethylformamide (DMF, Stanlab), and ethanol (EtOH, 96%, Stanlab). Graphene oxide (GO) was synthesised from graphite flakes via an improved version of Hummer’s method as reported in [68]: 1 g of fine graphite flakes was dispersed in 10 mL of concentrated phosphoric acid (85%). Afterwards, 5.5 g of potassium permanganate was added to the resulting suspension and stirred. In the next step, the mixture was cooled to 0 °C, and 100 mL of concentrated sulfuric acid (95%) was gradually added in small portions. The obtained slurry was kept at 60 °C for 12 h. After that time, it was centrifuged at 8000 rpm for 30 min; washed with a mixture of 70 mL of water, 35 mL of hydrochloric acid (35%), and 70 mL of ethanol; centrifuged again; washed with water until pH = 5; centrifuged one more time. The product was then washed with isopropyl alcohol and dried in an oven at 100 °C for 12 h.

### 3.2. Procedures of Syntheses

The detailed procedures for preparation of HKUST-1, Cur/GO, and the composites are given below. The assumed GO or rGO content in the composites was 10 wt.% considering the expected HKUST-1 yields of 50%. The latter is sensitive to the synthesis parameters (e.g., temperature, time, and pressure); hence, the amounts of DMF used in each procedure were adjusted to provide a 62% filling of the reactor’s volume.

#### 3.2.1. HKUST-1 (H1)

Copper nitrate (1.75 mmol) and H_3_BTC (1.75 mmol) were first dissolved separately in 35 mL of DMF each and then mixed in a Teflon-lined reactor. The reactor was heated up to 120 °C and, after 12 h, cooled down to room temperature. The obtained blue precipitate was filtered off, washed with DMF and ethanol, and dried at 80 °C for 12 h. The obtained sample of HKUST-1 was denoted H1.

#### 3.2.2. HKUST-1/GO Composites (H1/G)

**Figure 10:**
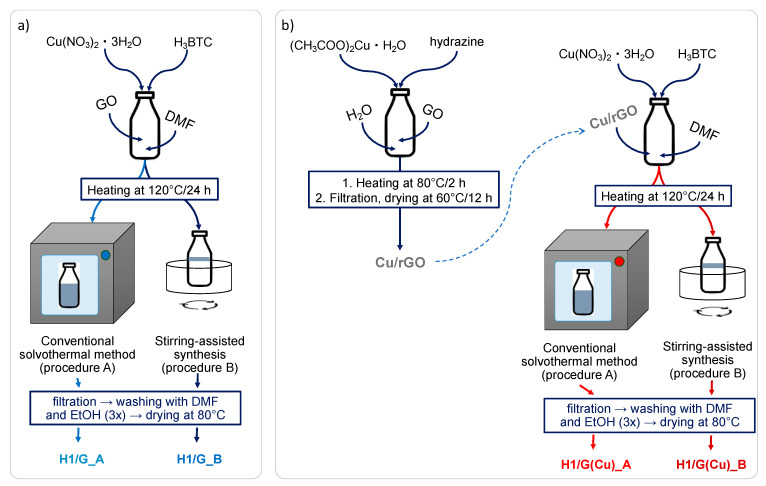
Synthesis schemes of GO-supported HKUST-1 (**a**) and Cu-decorated supported HKUST-1 (**b**).

An amount of 0.06 g of graphene oxide (GO) was suspended in 45 mL of DMF (during 10 min of sonication) and mixed with the solutions of copper nitrate (1.75 mmol dissolved in 35 mL of DMF) and H_3_BTC (1.75 mmol dissolved in 35 mL of DMF). The mixture was then placed in a Teflon-lined reactor and heated at 120 °C in an oven (procedure A), or oil bath under continuous stirring (procedure B). After 24 h the reactor was cooled down to room temperature and the obtained precipitate was filtered off under vacuum, washed with DMF and ethanol, and dried at 80 °C for 12 h. The HKUST-1/GO composites were denoted as H1/G_A (where A stands for conventional solvothermal method) and H1/G_B (where B refers to stirring-assisted synthesis). The scheme of synthesis is presented in Figure 10a.

#### 3.2.3. Cu/rGO

In the first flask, 0.1 g of GO was dissolved in 100 mL of distilled water and then sonicated for 10 min to obtain the GO suspension. In the second flask, 0.33 mmol of Cu(Ac)_2_ was dissolved in 30 mL of distilled water and then instilled into the GO suspension. The obtained mixture was heated up to 80 °C and 2.84 mL of hydrazine was added. After 2 h, the mixture was cooled down to room temperature. The synthesis of CuNPs supported on reduced graphene oxide (rGO) was carried out under continuous stirring. The obtained precipitate was filtered off under vacuum, washed with distilled water, and dried at 60 °C for 12 h. The nominal Cu content in the as-prepared material was 25 wt.%.

#### 3.2.4. HKUST-1/rGO (H1/G(Cu))

As is presented in Figure 10b, Cu/rGO (0.02 g) was added to DMF (15 mL) and sonicated for 10 min. Next, to the Cu/rGO suspension, the solutions of copper nitrate (0.5 mmol dissolved in 20 mL of DMF) and H_3_BTC (0.5 mmol dissolved in 15 mL of DMF) were added. The mixture was then placed in a Teflon-lined reactor and heated at 120 °C in an oven (procedure A), or in an oil bath under continuous stirring (procedure B). After 24 h, the reactor was cooled down slowly to room temperature. The precipitate was filtered off under vacuum, washed with DMF and ethanol, and dried at 80 °C for 12 h. The obtained samples were denoted H1/G(Cu)_A and H1/G(Cu)_B. The scheme of synthesis is presented in Figure 10b.

### 3.3. Physicochemical Characterisation of the Composites

The physicochemical properties of the obtained materials were analysed using powder X-ray diffraction (XRD), N_2_ adsorption/desorption at 77 K, scanning and transmission electron microscopy (SEM and TEM, respectively) with energy-dispersive spectrometry (EDS), Fourier Transform Infrared Spectroscopy (FTIR), and thermogravimetric analysis (TGA).

The XRD analyses were performed using a MiniFlex diffractometer (Rigaku, Japan, Tokyo) equipped with a Cu anticathode (λ = 1.54178 Å). The diffractograms were collected from 0 to 80° with a speed of 3°/min. The mean sizes of crystallites (D) for the selected diffraction planes hkl were calculated using the Scherrer equation [69,70].

The specific surface areas of the samples, total pore volumes, and the mean pore diameters were determined by N_2_ adsorption/desorption at 77 K performed using the Autosorb 3.01 (Quantachrome Instruments, Boynton Beach, FL, USA). Before the analyses, the samples were outgassed in vacuum at 140 °C for 24 h. The specific surface areas (S_BET_) were determined using the multipoint BET method, and the specific total pore volume (V_T_) was estimated from the N_2_ uptake at a relative pressure of P/P_0_ = 0.99.

The Fourier Transform Infrared Spectroscopy (FTIR) spectra were recorded on an IRAffinity-1S (Shimadzu, Japan, Kyoto) equipped with a Specac Quest ATR in the 400–4000 cm^−1^ range at a resolution of 1 cm^−1^ (256 scans).

The morphology of the samples was determined using a Cs-corrected S/TEM Titan 80–300 microscope (FEI Company, Hillsboro, Oregon, USA) operated at an accelerating voltage of 300 kV. The device was equipped with a Schottky-type field emission gun, CETCOR Cs-probe corrector, a Gatan Energy Filter, and energy-dispersive spectrometer (EDS) that allowed determination of the chemical composition of the studied samples.

The thermogravimetric analysis (TGA) was performed using a STA 409 PG Luxx apparatus by Netzsch, Selb, Germany (Mettler-Toledo, Columbus, OH, USA). The changes in sample mass vs. temperature were registered under air during the temperature increase from 25 to 800 °C with a rate of 10 °C/min.

## 4. Conclusions

The composites of HKUST-1 with graphene oxide, to be later used in catalytic reactions, were obtained using pristine GO and GO pre-decorated with CuNPs. The syntheses were conducted via the solvothermal method in static conditions and under continuous stirring. It was found that the type of graphene component used for composites preparation, as well as the parameters of synthesis, influenced the morphology, composition, and textural properties of the obtained products. The most homogeneous samples, consisting of the sandwich-type composite, were obtained with Cu/rGO, which provided Cu sites for direct crystallisation of HKUST-1 and its growth on graphene surface. The materials synthesized with pristine GO were less homogenous, especially the one obtained in static solvothermal synthesis. It was found out that for achieving a good yield of a sandwich-type HKUST-1/GO composite, the application of stirring is required as it affects the rates of nucleation and crystal growth of MOF by providing even heat distribution and mass transfer. In addition, stirring offers a good distribution of GO in the reaction mixture. In the case of HKUST-1 composites with Cu/rGO, the homogeneity of the produced material was achieved primarily through the presence of Cu sites, while the application of stirring had a less significant impact. As was confirmed by XRD, FTIR, and XPS, Cu was engaged in the formation of the connection between HKUST-1 and rGO, leading to the formation of a sandwich-type composite.

The performed research also revealed that the combination of HKUST-1 with GO or Cu/rGO had a negligible influence on the thermal stability of MOF. An insignificant decrease in thermal stability was observed when HKUST-1 and rGO formed a sandwich-like composite. Moreover, neither the addition of graphene material nor the application of stirring affected the mean size of HKUST-1 crystallites.

## Figures and Tables

**Table 1:** The mean sizes of HKUST-1 and Cu crystallites in obtained composites.

Sample	HKUST-1				Cu
	D _[200]_ (nm)	D _[220]_ (nm)	D _[222]_ (nm)	D _[400]_ (nm)	D _[111]_ (nm)
H1	48.0	56.0	52.9	48.1	-
H1/G_A	45.8	44.4	45.1	40.3	-
H1/G_B	49.1	51.0	49.5	44.3	-
Cu/rGO	-	-	-	-	17.8
H1/G(Cu)_A	50.1	52.7	50.3	44.9	-
H1/G(Cu)_B	45.1	42.6	43.3	35.5	-

**Table 2:** Contribution of bonds (in %) and Cu^2+^/Cu^+^ ratio in HKUST-1 and its composites with GO and Cu/rGO, calculated from the area under the peaks for the deconvoluted XPS C 1s, O 1s, and Cu 2p spectra.

	Bond	From C 1s			From O 1s			From Cu 2p
Sample		C=C	C-O	O-C=O	C-O-Cu	C=O	C-O-H	Cu^2+^/Cu^+^
H1		67.9	6.9	25.2	70.1	23.5	6.4	4.1
H1/G_A		61.1	25.7	13.2	91.5	0.0	8.5	1.6
H1/G_B		60.7	23.3	16.0	95.1	0.0	4.9	1.3
H1/G(Cu)_A		71.7	4.1	24.2	93.8	0.0	6.2	1.7
H1/G(Cu)_B		70.6	3.8	25.6	94.7	0.0	5.3	1.6

**Table 3:** Qualitative and quantitative composition of composites determined from TGA.

Sample	Wt.% of Component in the Sample			
	BTC^3−^ Linker	Graphene Component	CuO⟹Cu	
Cu/rGO	-	71.0	29.0	23.2
H1	40.0	0.0	60.0	47.9
H1/G_A	51.0	2.0	47.0	37.5
H1/G_B	49.6	7.2	43.2	34.5
H1/G(Cu)_A	52.7	8.1	39.2	31.4
H1/G(Cu)_B	61.6	7.6	30.8	24.6

**Table 4:** Textural properties of HKUST-1, GO, Cu/rGO, and HKUST-1/GO composites. S_BET_—specific surface area, V_total_—total pore volume, V_micro_—micropores volume, and d—mean pore size.

Sample	S_BET_ (m^2^/g)	V_total_ (cm^3^/g)	V_micro_ (cm^3^/g)	d (nm)
H1	666	0.46	0.26	2.8
GO	181	0.15	0.06	3.6
Cu/rGO	19	0.12	0.01	24.1
H1/G_A	804	0.45	0.27	2.3
H1/G_B	450	0.38	0.16	3.4
H1/G(Cu)_A	738	0.45	0.32	2.4
H1/G(Cu)_B	520	0.44	0.18	4.5

## Data Availability

The data presented in this study are available on request from the corresponding author.

## Sample Availability

Samples of the composites are available from the authors.
